# Nano-Conjugated Food-Derived Antimicrobial Peptides As Natural Biopreservatives: A Review of Technology and Applications

**DOI:** 10.3390/antibiotics12020244

**Published:** 2023-01-25

**Authors:** Brij Pal Singh, Rohit, K. M. Manju, Rohit Sharma, Bharat Bhushan, Sougata Ghosh, Gunjan Goel

**Affiliations:** 1 Department of Microbiology, School of Interdisciplinary and Applied Life Sciences, Central University of Haryana, Mahendergarh 123031, India; 2 Faculty of Applied Sciences & Biotechnology, Shoolini University of Biotechnology and Management Sciences, Solan 173229, India; 3 Amity School of Health Sciences, Amity University Punjab, Mohali 140306, India; 4 Department of Physics, Faculty of Science, Kasetsart University, Bangkok 10900, Thailand; 5 Department of Microbiology, School of Science, RK University, Rajkot 360020, India

**Keywords:** antimicrobial peptides, food safety, nano-conjugation, active food packaging, biofilm

## Abstract

In recent years, microbial food safety has garnered a lot of attention due to worldwide expansion of the food industry and processed food products. This has driven the development of novel preservation methods over traditional ones. Food-derived antimicrobial peptides (F-AMPs), produced by the proteolytic degradation of food proteins, are emerging as pragmatic alternatives for extension of the shelf-life of food products. The main benefits of F-AMPs are their wide spectrum antimicrobial efficacy and low propensity for the development of antibiotic resistance. However, direct application of F-AMPs in food limits its efficacy during storage. Therefore, the development of nanocarriers for the conjugation and distribution of potential AMPs may hold great potential to increase their bioactivity. This review highlights the significance of F-AMPs as a feasible and sustainable alternative to conventional food preservatives. The most recent developments in production, characterization, and mode of action of these AMPs against planktonic and biofilm forming pathogens are thoroughly discussed in this work. Moreover, nano-conjugation of F-AMPs with different nano-carriers and potential future application in food packaging are emphasized. This review may aid in comprehending the nano-conjugation of F-AMPs and offer insightful recommendations for further exploration and potential uses in the food processing industry.

## 1. Introduction

Maintaining life and nurturing good health depends on the availability of safe and nutritious food. Microbial food safety is therefore, the most crucial and challenging issue, along with balanced nutrition due to increased export of processed foods globally. The main factor contributing to food deterioration in both processed and unprocessed foods is microbial contamination. Unsafe food carrying bacteria, viruses, parasites, or chemicals causes more than 200 ailments, from cancer to diarrhea. According to the estimates, 600 million people worldwide (i.e., nearly one in ten) get sick after eating contaminated food, and more than 400,000 people die each year [1]. Additionally, it is anticipated that the annual cost of treating food-borne illnesses will be US$ 15 billion and that the overall cost is US$ 95.2 billion in productivity losses caused by foodborne illnesses, particularly in low- and middle-income nations [2,3]. Foods can become contaminated by microbes at many points during production, processing, and packaging. Due to this damage and spoilage, around 15 to 25 % of perishable food items remain unsafe for human consumption in the retail setting [4,5,6]. Moreover, increasing trends of antimicrobial resistance (AMR) in food-borne pathogens are one of the major factors limiting the safety and quality of processed foods. A number of these pathogenic bacteria are continuously acquiring AMR traits, posing a serious threat to human health and wellbeing [7,8]. Therefore, efforts are now being made to increase shelf life of processed food items while maintaining their safety, nutritional value, and sensory attributes through natural methods of preservation.

Antimicrobial peptides (AMPs) are presently being tested to enhance the quality and safety of food products. AMPs are low-molecular-weight proteins with antibacterial, antiviral, and antifungal activities. However, it is important to understand the difference between AMPs and food-derived antimicrobial peptides (F-AMPs) before looking into further details. While AMPs are host-defense peptides produced by many unicellular or multicellular organisms as a first line of defense against invading pathogens, the F-AMPs are peptides produced from food proteins in-vivo by gastrointestinal enzymes, or in-vitro through enzymatic hydrolysis, or during fermentation of food. The F-AMPs are essentially encoded into various food proteins and when released they primarily exert their antibacterial effects by disrupting bacterial cell membranes [9,10,11]. The basic structure of AMPs are abundant hydrophilic amino acids at the N-terminus, while non-polar hydrophobic amino acids are abundant at the C-terminus, which are crucial for AMPs to bind to bacterial cell membranes and alter their permeability to elicit antibacterial action [12]. AMPs have a number of benefits over conventional antibiotics, including the ability to positively influence the human immune response, a limited establishment of resistance, and broad-spectrum antibiofilm activity [13].

Despite being investigated as potential bio-preservatives, the effectiveness of F-AMPs has always been a concern due to their instability during food processing and storage, which may result in lower antimicrobial activity. Therefore, nano-conjugated F-AMPs have emerged as potential candidates against these constraints, along with an enhancement of their delivery through foods [14]. Moreover, the recent applications of these nano-conjugated AMPs in active packaging are also being explored, whereby the controlled release of AMPs enhances their efficacy and increases shelf life of coated, packaged food products. Antimicrobial packaging is a cutting-edge approach against proliferation of specific spoilage or pathogenic microorganisms.

Considering the importance of the above-mentioned aspects, this review details the current updates on the production of F-AMPs and their nanoforms to be used as nano-preservatives in active food packaging. It is crucial to highlight, that the term “nano-conjugation” in this review refers to the combination of AMPs with nanomaterials and encompasses any nanostructures including nano-capsulation, nanoparticles, nano-polymers, nano-liposomes, and nano-emulsions.

## 2. Food Safety Concerns and the Way Forward

Food provides a nutrient-dense conducive environment for growth of spoilage and pathogenic microorganisms. Foodborne infections are a catastrophic effect of pathogenic spoilage bacteria, and food loss due to deterioration at various stages of production through to consumption can be economically disastrous [4,15]. Food products can be contaminated in a variety of ways, resulting in deterioration of color, texture, and nutritional value as well as the growth of harmful bacteria rendering them inedible and unsafe. Bacterial contamination depends on the growth behavior of bacteria as living as sessile mass or as biofilms. Unlike planktonic or sessile bacteria as a contaminant in liquid food, bacteria can also adhere, colonize and form biofilms on to the surfaces of food ingredients, food processing equipment, and pipelines [16].

A biofilm is a functional consortium of microbes created mostly by exopolymeric substances (EPS) that can survive on abiotic surfaces such as plastic, glass, metal, and wood used in food processing equipment. As a result of their unique structure, biofilms increase the persistence of certain foodborne pathogens on product contact surfaces, making them more resistant to antimicrobial treatments [8]. The majority of foodborne infections are microbial biofilm-related and are considered an emergent public health problem worldwide [16].

There is a growing reliance of the population on the availability of the global food supply. Therefore, food safety is becoming increasingly important to people around the world. Food production and processing should be done carefully in order to maximize environmental and public health benefits [2]. Although several preservation strategies have been devised to prevent food spoilage, the problem persists. Consumers have found that the traditional methods (such as drying, freezing, heating, or salting,) for preserving food quality and safety over time, are not sufficient because recontamination occurs frequently, making the food unpleasant [5]. Modern processes such as chemical addition, irradiation, pasteurization, and canning, also produce similar outcomes. Therefore, food industries and food scientists are increasingly attempting to preserve food products using natural and green methods in response to rising consumer demands for healthier and safer diets. Natural antimicrobial agents have recently become one of the most reliant food bio-preservatives, along with a sophisticated encapsulation technology, which significantly reduces food deterioration and viability of spoilage and pathogenic microorganisms [17]. Moreover, the recent advancements in packing materials have made the natural ways of preservation more promising than the traditional physical or chemical methods of preservation. Antimicrobial packaging has recently received a lot of attention as a way to maintain food quality and shelf life, rendering it safer for human consumption. Antimicrobial packaging successfully imbeds the antimicrobials into the food packaging film material and then delivers it over a predetermined time period to kill pathogenic or spoilage bacteria thereby prolonging the shelf life by several times [18].

## 3. Recent Trends in the Production and Characterization of F-AMPs

AMPs derived from animal sources, such as milk and meat, are already being studied for their functional properties. However, the AMPs derived from plant proteins are gaining popularity as a more sustainable source. Since there has been a good number of recently published articles on production and characterization of bioactive peptides [10,11,19], the present review discusses only the recent developments in the field. The most common approach for producing bioactive peptides is enzymatic hydrolysis, with various advantages over other methods, including a shorter time of hydrolysis and processing parameters that are moderate and regulated. Protein hydrolysates and bioactive peptides may also be produced by starter and nonstarter microorganisms used in production of fermented dairy products, as these strains release extracellular proteolytic enzymes resulting in protein proteolysis [10,20,21]. Aside from that, a number of novel techniques are being investigated in an effort to improve the efficiency of current enzymatic hydrolysis processes, or to develop technological advances for the production of bioactive peptides. For instance, the generation of peptides using the ultrasound-mediated extraction method involves sending ultrasonic waves through a substrate. Furthermore, by promoting protein unfolding and enzyme interactions with the protein, high hydrostatic pressure (HHP) can be employed to enhance the enzymatic hydrolysis process. Similarly, by exposing the protein’s cleavage sites and hastening protein disintegration, microwave-assisted hydrolysis can be used to enhance protein hydrolysis. The generation of bioactive peptides is enhanced by pulsed electric field (PEF) processing, which denatures and unfolds proteins by rupturing hydrogen bonds and hydrophobic interactions [11,22]. After enzymatic hydrolysis, peptides must be purified from various food sources and characterized using various proteomics and mass spectrometry techniques. The Liquid Chromatography with tandem Mass Spectrometry (LCMS/MS) techniques have a very high resolution and separation efficiency and can recognize and classify complicated mixtures of peptides based on their molecular mass. In addition, in-silico methods are employed to generate known and unique peptide sequences from different samples. These bioinformatic methods can conceivably predict bioactive peptides as well, enabling researchers to concentrate on a small subset of peptide candidates with the highest potency of the desired activities [11,22]. Table 1 summarizes the AMPs derived from a variety of food protein sources using comprehensive techniques.

## 4. Antimicrobial Actions of F-AMPs

Most of the food borne pathogens exist in two phases of growth, the planktonic phase and as biofilms. AMPs have been tested for their efficacy against both types of growth behavior. The mechanisms of action of AMPs differ in these stages of growth.

### 4.1. AMPs Action against Planktonic Cells

**Figure 1:**
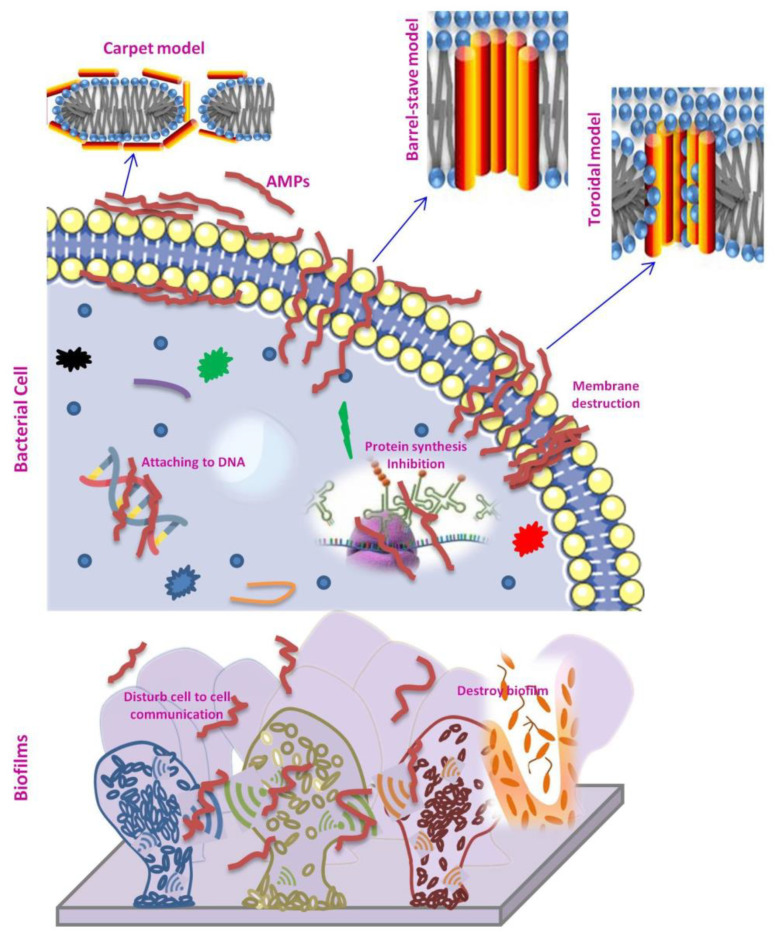
Mode of action of F-AMPs against bacterial cells and biofilms.

Although the exact mechanism of action of AMPs is still unknown, it has been suggested that these peptides interact on microbial cell membranes to cause pore formation and cell disintegration. Recent studies have however, identified additional potential mechanisms of action, such as interaction with particular intracellular targets, interference with bacterial metabolism, inhibition of protein and nucleic acid synthesis, disruption of the synthesis of cellular components, and inhibition of enzyme activity (Figure 1) [39]. Unlike antibiotics, these broad-spectrum activities of AMPs, therefore, prevent bacteria from developing resistance. In general, the AMPs share two physical characteristics: a cationic charge and a large number of hydrophobic residues. The majority of AMPs have secondary cationic amphipathic structures like α-helices and β-sheets, that enable them to interact with anionic bacterial membranes only through electrostatic interactions [40]. Moreover, the antibacterial activity of some specific amino acid residues in AMPs is largely correlated; for instance, AMPs with Arg and Val invariably have a significant antimicrobial impact because of their greater electrostatic film adsorption. Similarly, AMPs enriched with Pro are crucial for fungicidal activity as they regulate the mode of action of AMPs by crossing the cell membrane and interacting with intracellular macromolecules [12].

Cell membrane integrity is disrupted by the amphipathic nature of AMPs combined with the cationic nature of peptides. The negatively charged components of the cell membrane interact with the hydrophilic and cationic peptides. On the outer surfaces of gram-positive and gram-negative bacteria, teichoic acid and lipopolysaccharide (LPS) are present. Each of these compounds imparts a net negative charge to the surface, enabling the first electrostatic interaction with cationic AMPs. The hydrophobic domain of peptides, on the other hand, interact with the lipid bilayer to change its integrity. This causes the cell membrane to disintegrate, resulting in bacterial death [41,42,43,44]. The interaction of AMPs with cell membrane components could be divided into two categories, specific and non-specific interactions, depending on the requirements of cell surface receptors. For instance, Casein201, a human milk peptide, inhibited the growth of *Staphylococcus aureus* and *Yersinia enterocolitica* by disintegrating cytoplasmic structures and altering bacterial cell envelopes through non-specific electrostatic interactions [45]. These interactions are brought about by electrostatic contacts between the positively charged peptide moieties and the negatively charged components of the bacterial outer membranes; these electrostatic interactions do not need the presence of specific receptors at the bacterial membrane. Furthermore, peptides penetrate gram-negative bacteria’s outer membrane via hydrophobic interactions; the peptide may adopt a spatial conformation that facilitates the formation of a peptide–membrane complex, causing the outer membrane architecture to be disrupted, allowing additional peptide molecules to pass through [39]. Nisin, bacteriocins that preferentially bind to lipid II in the first step of its mode of action, is the first known receptor-mediated AMP which can be categorized as having specific interactions. At even nanomolar concentrations, this connection inhibits cell wall synthesis and causes pore formation, resulting in membrane permeabilization [46].

Generally, after the initial interactions, the AMPs typically build up at the surface and, after reaching a threshold level, they self-assemble on the bacterial membrane. At this stage, membrane active mechanisms of AMPs are demonstrated through three models: barrel-stave, toroidal, and carpet (Figure 1). The interactions of the hydrophilic portions of peptides cause AMP molecules to adsorb through the membrane surface and self-assemble in the barrel-stave model. The peptide bulk rotates perpendicularly to the plasma membrane when the laterally accumulated peptide monomers reach a specific density on the membrane. Finally, the peptide bulks are positioned along the bilayer hydrophobic portion, forming a channel with the hydrophilic surface facing inwards. Peptides are inserted perpendicularly in the bilayer in the toroidal model, similar to the barrel-stave model, but instead of peptide–peptide interactions, they create a peptide–lipid complex. This peptide-lipid conformation causes a local membrane curvature that is partially surrounded by peptides and partly by phospholipid head groups, resulting in the creation of a ‘toroidal pore’. The net arrangement of a bilayer in this model is differing from the barrel-stave in that the hydrophobic and hydrophilic arrangement of the lipids are preserved in the barrel-stave but disturbed in the toroidal model. This gives the lipid tail and lipid head groups different surfaces to interact with. Because the pores disintegrate quickly, some peptides translocate to the inner cytoplasmic leaflet, where they reach the cytoplasm and may target intracellular components. AMPs, on the other hand, are bonded parallel to the membrane surface in the carpet model. The peptides accumulate until a threshold concentration, at which they reorient towards the inside of the membranes and form micelles with a hydrophobic center that results in membrane breakdown. The carpet model does not require precise peptide–peptide interactions between membrane-bound peptide monomers, nor does it require that the peptide be inserted into the hydrophobic core to produce transmembrane channels or unique peptide structures [19,43,46,47,48].

Beside membrane-active mechanisms, various AMPs have recently been identified that target essential cell components and cellular activities, resulting in bacterial death. These AMPs pass through the cell membrane without disturbing it, and then interact with intracellular targets to obstruct vital cellular activities. The proline-rich peptides have intracellular activity through suppression of bacterial protein synthesis. For example, Bac7 from bovines interacts with the ribosome and inhibits translation by impeding the transition from the initiation to the elongation phase [46,49]. The peptide αs165-181, which is derived from αs2-casein of ovine milk, exerts antibacterial effect through destruction of the bacterial cell membrane and attachment to their genomic DNA [23]. AMPs, like conventional antibiotics such as penicillin, are also reported to block cell wall synthesis. However, AMPs are interacting with essential precursor molecules that are essential for cell wall synthesis, rather than binding with particular proteins involved in the synthesis of cell wall components as reported for antibiotics [43].

### 4.2. AMPs against Bacterial Biofilms 

Pathogens contaminate food during processing conditions, whereby the pathogens come into contact with the surface of food itself and the related equipment. After adhering to the surface, most of the pathogens form biofilm on solid or viscous food surfaces and abiotic surfaces of equipment. A biofilm is formed via multiple processes, starting with adherence to biotic or abiotic surfaces, which leads to the establishment of a micro-colony, which then gives rise to three-dimensional structures, and finally, elimination after maturation of biofilms. Microorganisms are embedded in a biofilm by a matrix of EPS that acts as a barrier and make the cells resistant to a variety of hostile environments such as sanitizers, disinfectants, antibiotics, and other hygienic conditions, posing a challenge to the food industry in maintaining quality and safety of foods [8,50,51].

The antibiofilm effects of AMPs have been studied in recent years; Batoni and coworkers proposed two modes of action to explain the antibiofilm activity of AMPs, namely classical and non-classical mechanisms. The classic mode of action relies on known bactericidal effects of AMPs on planktonic bacteria that limit their ability to form biofilms. The non-classical process is linked to an AMP activity that targets the biofilm fundamental characteristics [52]. AMPs may block cell–cell interaction by binding to the bacterial surface, limiting the bacterial adherence to the biomaterial surface, interfering with cell communication signals, or promoting the down regulation of genes required for biofilms development [46]. Biofilms’ cell signaling systems can also be disrupted by AMPs (Figure 1). The peptides reduce biofilm development by inhibiting RNA synthesis and blocking the synthesis of guanosine tetraphosphate and pentaphosphate via the enzymes RelA and SpoT. Additionally, AMPs can function as quorum sensing inhibitors (QSI), which impede cell-to-cell communication and prevent the formation of new biofilms. For instance, *Listeria monocytogenes* biofilm development was reduced using the bacteriocins, lactocin AL705, which inhibited quorum sensing (QS) by inactivating the signal molecule autoinducer-2 (AI-2) [53]. The QS, a highly organized cell-to-cell signaling communication system, controls bacterial population density by regulating the synthesis of virulence factors in response to variations in bacterial population density [54]. The signal molecules such as autoinducer, N-Acetylated-l-homoserine lactones (AHLs) and peptide-based signal molecules play the key role in QS [55].

## 5. Nano-Conjugation of F-AMPs

Despite the numerous benefits of AMPs there are still obstacles in attaining their full potential owing to their sensitivity to temperature and pH, as well as to gastrointestinal digestion after oral administration. The direct application of F-AMPs into foods has limited benefits as they may be partially inactivated or neutralized, and are easily dispersed when entering the food matrix. It is critical to preserve the peptides against inactivation and to manage distribution selectively. With the rapid development of nanotechnology in recent years, it is possible to design a suitable delivery system that can effectively improve the absorption and tailored release of peptides or drugs, which is important for enhancing their bioavailability and bioactivity. F-AMPs can be effectively shielded from the environment by using nano-technology methods. Peptide environmental exposure is reduced, and their aqueous solubility, bioavailability, circulation time, and cellular uptake are all enhanced with the use of these methods. Nanopolymers, metallic nanoparticles, nanoemulsions and liposomes are commonly designed to conjugate, encapsulate, safeguard, and to control the release of bioactive compounds that can be used with F-AMPs [56,57,58]. Some examples of bacteriocins’ (AMPs from food-grade bacteria) conjugation with nanomaterials have been discussed in this section due to the paucity of data on the nano-conjugation of F-AMPs. It is anticipated that similar techniques may also be used for F-AMPs.

### 5.1. F-AMPs with Polymer Nano-Conjugates

In order to protect AMPs from different processing conditions and to increase their efficacy and bioactivity, they have been conjugated with polymers and delivered via nano carrier systems. Several polysaccharides such as starch, pectin, cellulose, dextrin, gum, alginate, chitosan, and cyclodextrin, have been exploited as carriers of food components during production and processing (Table 2) [58]. It is a sustainable and secure alternative to generate polymer nanoparticles from natural sources for food applications. Since metal toxicity and bioaccumulation are constant constraints with metal based nanomaterials, natural polymer-based antimicrobial nano-conjugates have benefits over metallic nanoparticles in food packaging and food preservation. Moreover, the increased area/volume ratio that occurs when biopolymers are converted into nanomaterials might enhance or intensify their natural characteristics [59].

AMPs are dissolved, trapped, encapsulated, or conjugated into a matrix in AMP nano-polymers, which are constructed from biocompatible and biodegradable polymers with sizes ranging from 10 to 1000 nm. There are two basic phases involved in the synthesis of polymer nanoparticles: first, the formation of an emulsified system; and second, the preparation of nanoparticles either via precipitation or polymerization of monomers or gelation of a polymer. Polymer-based nano-conjugates can easily be used in different perspectives for controlled delivery of target bioactive molecules. Biopolymers are dissolved in the appropriate solvents to generate film-forming solutions; for instance, gelatin and alginate with an extremely hydrophilic nature are easily soluble in water, whereas chitosan with a distinctive cationic structure can only dissolve in acidic solutions. The AMP solution is added when biopolymers have entirely dissolved in the solvents. The resulting film-forming solutions are subsequently cast onto flat plates after being degassed. The solvents are dried for a few hours to allow them to evaporate. Stable three-dimensional film networks are produced as a result of this process, which also establishes the main intermolecular connections between various film components [60]. The polymer nanoconjugates deliver the AMPs to the target site in one of three ways: (1) by hydration-induced swelling of nano-conjugates, followed by release through diffusion; (2) by an enzymatic reaction that causes the polymer to rupture, cleave, or degrade at the site of delivery; or (3) by dissociation of the drug from the polymer and its de-adsorption/release from the swollen nano-conjugates [61,62]. However, this article will not delve into the specifics of how nano-conjugates and nanoparticles are prepared, as it has already been discussed elsewhere [63,64]. Instead, this paper presents current research on the efficacy of nano-conjugated AMPs in food preservation.

**Table 2:** Nanomaterials used and methods of preparations of polymer and metallic nano-conjugates.

Type of Nano-Conjugates	Nanomaterial Used	Methods of Preparations	Reference
Polymer nano-conjugates	Chitosan Alginate Gelatin Albumin Poly(lactide) Poly(lactide-co-glycolide) Poly(epsilon-caprolactone) Poly(isobutylcyanoacrylate) Poly(isohexylcyanoacrylate) Poly(n-butylcyanoacrylate) Poly(acrylate) and poly(methacrylate) Poly(lactide)-poly(ethylene glycol) Poly(lactide-co-glycolide)-poly(ethylene glycol) Poly(epsilon-caprolactone)-poly(ethylene glycol) Poly(hexadecylcyanoacrylate-co-poly(ethylene glycol) cyanoacrylate)	Solvent evaporation Nanoprecipitation Emulsification/solvent diffusion Salting out Dialysis Supercritical fluid technology Particle replication in non-wetting templates Interfacial polymerization Controlled/Living radical polymerization Ionic gelation or coacervation of hydrophilic polymers	[61,62]
Metallic nano- conjugates	Aluminum Cerium Copper Gold Iron Manganese Nickel Platinum Silica Silver Thallium Titanium Zinc	Thermal decomposition method Sol–gel method Hydrothermal and solvothermal method Microwave-assisted method Polyol method Sonochemical method Liquid–liquid interface method Phase-transfer method Biosynthesis method Template-directed synthetic method	[65,66,67]

Among different biopolymers, chitosan is a widely used nanomaterial for food applications. Chitosan is a non-toxic cationic polysaccharide produced by deacetylating chitin from the exoskeletons of crustaceans. Chitosan is reported to have antibacterial activity and can disrupt bacterial cell walls and cause cell lysis in gram-negative bacteria by binding to outer membrane protein A and lipopolysaccharide at a neutral pH. Lactoferrin was recently conjugated with chitosan and gellan, utilizing electrostatic complexation, in order to improve its antibacterial characteristics. Fresh strawberries coated with the conjugate, which had a MIC of 0.0117 mg/mL, effectively preserved their physicochemical qualities. The improved antibacterial effect of the conjugate may be due to synergistic action of lactoferrin and chitosan [59]. Moreover, milk-derived bioactive peptides (caseinophosphopeptides), gallic acid and chitosan were combined to form physicochemically stable nanoparticles. Strong antioxidant activity and cytotoxicity against Caco-2 colon cancer cells were displayed by the nanoparticles. Under simulated gastrointestinal conditions, the nanoparticles also demonstrated improved delivery properties that prevented their degradation in neutral and alkaline environments [68]. Recently, zein-egg white derived peptide–chitosan nanoparticles were successfully created by spontaneous assembly to improve the stability and bioactivity of curcumin. The nanoparticles have a strong encapsulation efficiency for curcumin and are typically nano-spherical in structure. This study supports the hypothesis that bioactive peptides/AMPs obtained from food could serve as a perfect carrier for the administration of hydrophobic nutraceuticals [69].

In another study, chitosan-based film was impregnated with silicon dioxide nanoparticles and nisin for preservation of blueberries. After treatment, the nano-conjugate significantly prevented the growth of both molds and the mesophilic microbial population, and controlled the shrinkage and decay rates of blueberries by 38.52% and 8.61%, respectively [70]. Similarly, the combination of nisin, nano-silica, and chitosan films significantly enhanced the shelf-life of the edible mushroom, *Agaricus bisporus*. Initially, 1% nano-silica was blended in a chitosan solution which was further supplemented with 1% nisin. The resulting composite was then coated onto the mushrooms using a dipping process. The levels of reactive oxygen species such as hydroxyl radicals, superoxide anions, and hydrogen peroxide were significantly lower in the treated mushrooms indicating a longer storage life with negligible free radical mediated damage [71].

Furthermore, a coating developed using gelatin, thymol and nisin was effective in maintaining the chemical quality indices of rainbow trout fillets during 16 days of storage at 4 °C. The nanocomposite treated sample showed a pH of 6.18 after storage for 16 days, unlike the untreated group (pH = 7.12). It was speculated that bacterial metabolism resulting in food spoilage is associated with the release of ammonia and/or other alkaline products that results in an increase in the pH. The lower pH in the treated group was indicative of significant bacterial inhibition on the food sample [72]. In another study by the same group, the composite coating showed a significant reduction in total viable bacteria, total psychrophilic bacteria, and hydrogen sulfide producing bacteria, lactic acid bacteria and *L. monocytogenes* counts. Hence, it was evident that both nisin and thymol on impregnation in gelatin based nanocoatings, can help in maintaining the chemical quality of food samples, and thereby increase the shelf life of food [73].

Hydrogel beads or microgels, a network of one or more types of biopolymers cross-linked by physical and/or chemical linkages, are also utilized to conjugate bioactive peptides. Microgels are promising delivery vehicles for encapsulating, shielding, and releasing bioactive peptides. As food-grade biopolymers, proteins and/or polysaccharides are frequently employed in the synthesis of microgels for use in food applications. Using various production techniques, bioactive peptides can be captured inside microgels either before or after microgel formation [58,63,64].

Synthetic polymers like polyethylene glycol (PEG), poly-l-lysine (PLL), and poly lactic-co-glycolic acid (PLGA), and others, are also commonly used in drug delivery, where they act as drug carriers across bacterial membranes before dissolving and releasing the drug at its specific target site once inside the cell membrane [74,75] (Table 2). PEG is a non-toxic, non-immunogenic, FDA-approved polymer that improves the biocompatibility of a wide range of substances. The goal of conjugating AMPs with PEG is to prevent them from being recognized and degraded by proteolytic enzymes, as well as to expand the size of the AMP [76]. Furthermore, PLGA, polyester made of lactic acid and glycolic acid, has been given FDA approval for use in numerous medicinal products because it is easily biodegradable. Synthetic polymers can be specified more precisely than natural polymers, can be produced at a lower cost on a large scale, and are more stable during storage [77].

### 5.2. Metallic Nano-Conjugates of F-AMPs

Metallic nanoparticles (MNPs) produced from noble metals, such as gold and silver, have also been reported as nano-carriers for AMPs (Table 2). Most MNPs adhere to the surface of bacterial membranes by electrostatic interactions due to their vast surface area and surface charge, and disrupt the membrane’s integrity. Noble metals are resistant to oxidation and corrosion, making them suitable for nanoparticles formation and reduced toxicity [76]. However, when dealing with metal nanoparticle synthesis, the approach chosen must be straightforward; less expensive, environmentally friendly, commercially feasible, and simultaneously with a manageable particle size, shape, and homogeneity. Since the nanoparticles are kinetically unstable and need to be protected from aggregating into larger particles, micelles, polymers, and coordinative ligands are widely utilized as stabilizers of nanoparticles. Typically, solution-based nanofabrication techniques impart more control and reproducibility to MNPs. There are many different nanofabrication techniques described in the literature, including precipitation, deposition precipitation, sol–gel, liquid–liquid interface technique, hydrothermal and solvothermal syntheses, microwave-assisted processes, polyol method, template-directed synthesis, and ionic-liquid assisted methods (Table 2) [65].

Recently, Al-hadede and Hassan, conjugated an AMP enterocin on silver nanoparticles (AgNPs) using *Alettaria cardamomum* extract. The enterocin–AgNPs conjugate showed significant enhancement of antimicrobial activities against *Escherichia coli*, *Pseudomonas aeruginosa*, *Salmonella typhimurium*, *Bacillus subtilis*, and *Staphylococcus aureus* in comparison to non-conjugated metallic nanoparticles. A similar trend was also noted against the yeast *Candida albicans*, where around 45% enhancement of activity was reported after enterocin conjugation [78]. In a similar study, enterocin-capped AgNPs (En-AgNPs) were fabricated for inhibiting food-borne pathogens. A two- to sixteen-fold higher inhibitory activity was observed against *Pediococcus acidilactici* LB42, *E. coli* ATCC 25922, *Bacillus cereus*, *Listeria monocytogenes*, *Micrococcus luteus*, *Shigella flexneri*, and *S. aureus* compared to the citrate capped AgNPs as determined by MIC values. The combined effect of enterocin and the AgNPs resulted in significant loss of the membrane integrity and bacterial cell death [79]. The synergistic interaction between AMPs and nanocarriers may be responsible for the improved antimicrobial effect of these nano-conjugates.

In another study, a pediocin conjugate (AuNPs, Pediocin–LAP (Listeria adhesion protein)) showed a 31% higher reduction in *Listeria* counts in biofilms during a prolonged incubation of 48 h, confirming a remarkable antibiofilm activity. Bacteriocin (Bac4463 and Bac22 from *Lactobacillus herbarum*) capped AgNPs showed significant inhibition against *S. aureus*, *P. aeruginosa*, *S. flexneri* and *B. cereus* that was almost 1.5 to 2.3 folds higher than their free counterparts. The MIC for Bac4463-capped AgNPs and Bac22-capped AgNPs were 8 μg/mL and 2 μg/mL against *S. aureus*, and *S. flexneri*, respectively [80].

In a similar effort, the activity of plantaricin (an antimicrobial from *Lactobacillus plantarum* strain) was enhanced through conjugation with AgNPs. The nano-conjugate showed higher antibacterial activity against *E. coli, S. aureus, P. aeruginosa, S. paratyphi B, S. faecalis, B. cereus*, and *L. monocytogenes*. The MIC and MBC values of the plantaricin–AgNPs nanocomposite were 0.004 mg/mL and 0.625 mg/mL, respectively, against *S. aureus*. It is interesting to note that the stability of the bacteriocin at 4 °C increased from 5 days to 60 days after conjugation with the AgNPs [81]. Such a notable enhancement of the antimicrobial activity was attributed to the large surface area of the nanocomposite with a smaller dimension, which in turn enhanced their permeability through the plasma membrane, facilitating their entry. The outer cell boundary of the microbes is negatively charged which can attract positively charged metal nanoparticles. The latter then release ions that interact with the thiol group (-SH) of the proteins that are associated with the transporters that impairs the permeability of the cellular metabolites and disrupts the transport of the nutrients, eventually resulting in cell death. AgNPs can further affect bacterial DNA and disrupt its multiplication and gene expression of proteins and enzymes necessary for ATP production and other vital cellular metabolism [81].

### 5.3. F-AMPs Liposomal Nano-Conjugates

Liposomes are spherical vesicles containing at least one lipid bilayer, that have been widely studied for drug delivery. Phospholipid bilayers with hydrophobic and hydrophilic nano-order surfaces make up liposomes. These can bind, transport, and release bioactive peptides that are both hydrophilic and lipophilic due to their amphipathic structural characteristics. Hydrophobic substances are confined in the bilayer membrane, whilst hydrophilic molecules are trapped in the aqueous core [58]. These have been shown to increase bioactive molecule delivery by acting as circulating micro-reservoirs for long-term release. In a similar manner, soy lecithin-derived nanoliposomes were successfully used to encapsulate whey peptides with a range of molecular weights. Among the various factors which affect encapsulation effectiveness is the net charge on peptides rather than their molecular weight. Anionic peptides were reported to have lower encapsulation efficiencies than cationic peptides; this is likely because the anionic peptides were electrostatically attracted to the liposome surface [58,82,83]. In one study, liposomes encapsulating AMP pediocin were produced using different phosphatidylcholine concentrations in the microfluidizer. The production of the pediocin-encapsulating liposomes with a size of 144 nm was caused by the addition of 3 and 5 percent phosphatidylcholine 500 and 1000 bar of pressure, and 1 cycle of microfluidization. Zeta potentials less than 20 mV suggested that the phospholipid bilayer might agglomerate, flocculate, sediment, or tear [84]. In another study, fabrication of chitosan stabilized nano-liposomes, termed chitosomes, was reported to enhance the controlled release and antimicrobial effect of nisin-Z against multidrug resistant foodborne pathogens. The chitosomes were synthesized using soy lecithin, nisin, chitosan and 1% tripolyphosphate as cross-linker under stirring conditions followed by ultrasonication. The encapsulation efficiency of nisin loaded nano-liposomes was 56.53% with a loading capacity of 40%. The interaction between chitosan and liposome was mostly via amide I and amide II groups, while between nisin and chitosan it was due to a weak hydrogen bonding. This facilitated more nisin encapsulation between lipid and polymer layer, as well as between the meshwork of chitosan. The diameter of the nisin loaded liposome was 80 to 108 nm, which might be attributed to the nisin encapsulation in the core. The chitosomes significantly inhibited the pathogenic bacteria *S. aureus, E. faecalis* and *L. monocytogenes* [85]. Nanoliposomes of lactoferrin were developed by Guan and colleagues which possessed satisfactory stability at 37 °C for 4 h. Both free lactoferrin and lactoferrin nanoliposomes had a dose-dependent impact on the survival of Caco-2 cells, with lactoferrin nanoliposomes having a more pronounced effect [86]. Several advantages of liposome-based nanovesicle systems include self-assembly, amphipathicity, low toxicity, flexibility, protection against premature breakdown or inactivation, adjustable biophysical and physicochemical properties, and biocompatibility. However, due to their great sensitivity to their environment, frequent breakdown in food matrices and gastrointestinal environments, and instability issues during oral distribution, liposomes are limited in their therapeutic application. This problem is typically resolved using the appropriate lipid mixture, polymer coating, double liposome creation, proliposomes, and insertion of stabilizing lipids in the structure [58].

### 5.4. Nanoemulsions of F-AMPs

Nanoemulsions are colloidal dispersions made up of two immiscible liquids, usually water and oil, with one of the liquids dispersed as tiny droplets in the other. Nanoemulsions are oil, water, and surfactant-based, small, evenly sized droplets with high kinetic stability and low viscosity. These are frequently utilized in the nanoencapsulation of lipophilic and hydrophilic bioactive substances for food, medicine, and pharmaceutical applications. Nanoemulsions-based delivery systems, typically used to administer bioactive substances orally, can be created by microfluidization, ultrasonication, solvent diffusion, homogenization, or a phase-inversion temperature approach. This system has less sedimentation, phase separation, or creaming events due to their great kinetic stability. Additionally, this system improves the fraction of compounds that reach a target, boost the stability, water solubility, and bioavailability of bioactive compounds, and lower the toxicity connected with off-target delivery [58,63]. This colloidal system can also be divided into oil in water (O/W) or water in oil (W/O) types depending on how closely the oil and water phases are spaced from one another. However, bioactive proteins, may stick to the surfaces of oil droplets, whereas O/W emulsions are insufficient for encapsulating proteins because the interiors of the oil droplets are too hydrophobic [63]. A novel chitosan based nanoemulsion coating containing *Ziziphora clinopodioides* essential oil and nisin was developed for effective inhibition of *E. coli* O157:H7. The count of *E. coli* O157:H7 was reduced by around 50% on the 16th day after treatment with the nanoemulsion. This indicated their promising application as food preservatives and for prevention of food spoilage [87]. However, proteins present at the outside of oil droplets may not be protected from breakdown when exposed to food or GIT conditions, and adsorption of proteins to surfaces can modify their structure and activity due to surface denaturation. In order to better protect the protein-coated droplets, additional forms of colloidal particles, such as biopolymer microgels, may be used to enclose them. An alternative method is to electrostatically deposit bioactive proteins onto the emulsifier-coated oil droplet surfaces, where they can form one or more layers. To modify the protein layers’ encapsulation, protection, and release qualities, additional biopolymer layers can be placed on top of them [63].

Solid lipid nanoparticles are another type of colloidal system, which are often made by heating an oil in water nanoemulsion to a temperature beyond the oil phase’s melting point, then cooling the mixture to encourage the crystallization of the oil droplets. This system consists of an emulsifier that provides stability in aqueous solution and a lipid core made up of fatty acids, waxes, steroids, triglycerides or partial glycerides. The synthesis of peptide-loaded solid lipid nanoparticles can be accomplished using coacervation, solvent emulsification evaporation, microemulsion, solvent emulsification diffusion, supercritical fluid technology, or high-pressure homogenization. Solid lipid delivery systems are stable, nontoxic, amphipathic delivery systems that can encapsulate, safeguard, and transport both hydrophilic and lipophilic bioactive peptides, much like liposome- and nanoemulsion-based delivery systems. Bioactive peptides produced from whey, fish protein, and antimicrobial peptides have all been encased in solid lipid nanoparticles [58,63]. A recent study reported the preparation of solid lipid nanoparticles using two fractions of peptides generated from oat globulin. With comparable encapsulation efficiencies, zeta potentials, and storage durability, both fractions were tightly sealed. In simulated gastrointestinal fluids (SGF), the release and degradation rates of the encapsulated peptide fractions varied, but both fractions were resistant to subsequent hydrolysis and retained high bioactivity rates [88].

## 6. Applications of Nano-Conjugated F-AMPs in Active Food Packaging

The consumers’ demand for more natural products has compelled food manufacturers to test novel processes/ technologies for food preservation. The preservatives have been utilized in food formulations or packaging to extend the shelf life of foods, with natural agents being the most investigated due to customer demand for natural ingredients in food. Food packaging plays an important role in ensuring the quality and safety of food products till they reach the ultimate customers. Natural antimicrobials can be used as preservatives in two ways: directly in the food composition or by including them in the packaging structure or coating materials, known as active packaging. The antimicrobials that are directly incorporated into food have a lower biological activity against bacteria due to their diffusion across the food matrix. These are also sensitive to harsh processing and storage conditions, such as high temperatures, which can substantially impair their antimicrobial efficiency. As a consequence, the encapsulation/conjugation approach is one of the most successful methods for designing delivery systems for natural antimicrobials with improved functionalities. Active packaging materials incorporating AMPs appear to be a potential technique for reducing food deterioration and increasing food safety and shelf life. The use of packaging materials impregnated with antimicrobial agents, rather than their direct integration, is expected as a more efficient method of food preservation. Foodborne pathogenic and/or spoilage bacteria are prevented or inhibited by antimicrobial packaging, which interacts with the food surface or the headspace inside the package. Controlled diffusion of antimicrobials from packaging materials to food surfaces may not only delay or prevent the initial growth of unwanted microbes on food surfaces, but also provide residual action that lasts throughout food storage and distribution to the final consumers [6,17,39].

The use of F-AMPs as an antimicrobial agent in active packaging is a viable option that has a number of advantages, including the elimination of chemical preservatives, the reduction in food losses due to spoilage, and the development of health-promoting dietary supplements [14]. Several AMPs are being investigated for their ability to inhibit foodborne pathogens in a variety of food matrices, including dairy products, meat, fruits, and beverages [42]. Recently, Ranjith et al. investigated the antifungal efficacy of edible coatings made with bioactive peptides derived from palm kernel cake fermentation. The edible coating produced by integrating bioactive peptides in chitosan prevented the growth of fungi such as *Colletotrichum gloeosporioides* and *Botryodiplodia theobromae* in mangoes [89].

**Figure 2:**
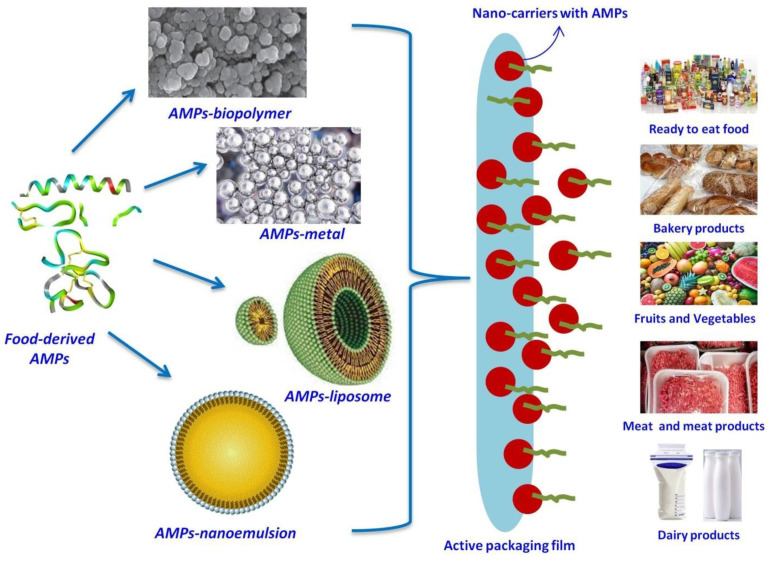
Nano-conjugation of F-AMPs with diverse nanomaterials and potential application in active food packaging of different food products.

F-AMPs are delivered to foods in six different ways by nano-conjugated packaging: (1) AMPs coatings are applied to foods via head space, (2) polymers are applied to foods via head space, (3) coatings are applied to foods directly, (4) AMP pads are applied to foods via head space, (5) AMP pads are applied to foods directly, and (6) AMP impregnated edible coatings are applied to foods directly [14]. The knowledge of AMPs diffusivity both inside and outside the packaging film is important because the efficacy of antimicrobial films is dependent on the migration of active substances. Along with molecular weight, polarity, solubility, and affinity of AMPs, diffusivity is influenced by the packaging atmosphere (pH, water activity, and temperature), and pore size, polymeric chain flexibility, polarity, and packing density of the polymer structure [90]. In an intriguing study, a bactenecin-derived peptide 1018K6 was covalently conjugated to a polyethylene terephthalate (PET) matrix in order to fabricate a packaging material with antibacterial effects; when tested with mozzarella cheese, it drastically reduced yeast and mold populations within the first 24 h [5]. AMPs have also been successfully loaded into nanocarriers alongside other bioactive substances. For instance, nisin was successfully loaded into soy soluble polysaccharide-based nanocarriers along with curcumin, resulting in both an antibacterial as well as an antioxidant bioactive ingredient [3,91]. A recent study found that CuO nanoparticles stabilized with gelatin had a great potential for use in food packaging, both as an independent nanofilm and as a component of other packaging materials [92]. In a similar vein, F-AMPs may be fabricated into packaging films in conjunction with polymers, nanoparticles, or other nanomaterials. It is possible to create nano-conjugated F-AMPs in food packaging by incorporating them into biopolymers and packaging materials. These materials are then placed in the head space of the packaging, where they interact with the food and release the active ingredients during the course of storage. Additionally, AMP-based edible coating can be a good choice for direct application in fruits and vegetables (Figure 2).

## 7. Conclusions and Future Outlook 

Given the growing demand for safe, high-quality and sustainable foods, natural antimicrobial substances such as F-AMPs are a subject of great interest in the field of food science. In addition to enabling regulated release of F-AMPs, the usage of nanostructures offers an intriguing alternative for protecting and delivering antimicrobials with higher efficacy in food. AMPs can be trapped by a variety of nanostructures, maintaining the stability of compounds that are otherwise sensitive to the circumstances of food processing and storage. Despite the enormous potential of nano-conjugated F-AMPs, only a small number of applications for food packaging have been studied. Therefore, more research should be done on developing F-AMPs using various nano-carriers and studying them in actual food packaging systems. Moreover, novel AMPs from various food sources and their nano-carrier combinations may be tested to improve the effectiveness of food preservation and shelf-life. Furthermore, the impact of specific food processing parameters, such as temperature and pH, on AMP activity along with investigations on the behavior of AMPs within complex food systems are desperately required. Additionally, it is also important to assess the impact of F-AMPs on the sensory and qualitative attributes of food. Apart from that, before being used in food, extensive safety studies of nano-conjugated F-AMPs in in-vivo and human models must be performed. It is also advised to have a thorough understanding of the molecular principles governing the function of F-AMPs in order to establish better interpretations. The relevant regulatory and approval protocols must be used, however, for the incorporation of nano-conjugated F-AMPs in food packaging and food coating applications. Based on the approaches described in this review, which employ various strategies in the nano-conjugation of F-AMPs, we can anticipate an increase in AMP-based packaging of food products capable of circumventing food spoilage and drug resistance over the next decade. We predict that development of nano-conjugated AMPs will expand beyond the scope of targeting food-borne pathogens.

## Figures and Tables

**Table 1:** List of important antimicrobial peptides obtained by proteolysis of food protein and their main properties.

Source of Peptide	Type or Sequence of Peptide	Effect	Reference
Ovine milk	αs2-casein (αs165-181) peptide	Antimicrobial effect against *E. coli*, *S. aureus*, *B. subtilis*, *L. monocytogenes*, *B. cereus*, and *S. enterica serovar Enteritidis* with MIC 3.9 mg/mL for *E. coli* and 7.8 mg/mL for other bacteria	[23]
Bovine milk	TKLTEEEKNRLNFLKKISQRYQKFALPQYLK	Inhibits the growth of *B. subtilis* and *E. coli*, with MIC value of 4.0 µM and 16.2 µM, respectively	[24]
Buffalo casein	YLGYLEQLLRLK	Antimicrobial against *E. coli*, *S. aureus*, *L. monocytogenes* and *S. typhimurium* at concentrations ranging from 0.8 to 1.6 mg/mL	[25]
Chickpea protein	RIKTVTSFDLPALRFLKL, RIKTVTSFDLPALRWLKL	Antimicrobial activity against a variety of bacteria, showed MIC down to 15.6 µmol/L	[26]
Rice bran proteins	LRRHASEGGHGPHW, EKLLGKQDKGVIIRA, SSFSKGVQRAAF	Antimicrobial and lipopolysaccharide (LPS)-neutralizing activities	[27]
Soybean meal	HTSKALLDMLKRLGK	MIC of 72.5 and 72.5 μM against *Vibrio alginolyticus* and *V. parahaemolyticus*, respectively	[28]
Bovine αs2- casein	KTVYQHQKAMKPWIQPKTKVIPYVRYL	Effective against gram-positive and gram-negative bacteria	[29]
Bovine αs2- casein	YYQQKPVA	Effective against gram-positive and gram-negative bacteria	[30]
Bovine κ-casein	VQVTSTAV	Antimicrobial effect against gram-positive bacteria.	[30]
Bovine κ-casein	PAAVRSPAQILQ	Antimicrobial effect against gram-positive and gram-negative bacteria	[30]
Milk	αS2-Casein f (183–207)	Antimicrobial activity against *Cronobacter sakazakii* and *Listeria monocytogenes*	[31]
Edible insect Musca domestica	Md-AMPs	Improves the shelf-life of chilled pork by up to 6 days and exhibits excellent activity limiting microbial growth by preventing DNA synthesis	[32]
Turbot viscera	GITDLRGMLKRLKKMK	Inhibits the growth of *E. coli*, *S. typhimurium*, *S. aureus*, *L. monocytogenes*, *B. subtilis*, and *H. alvei*	[33]
Slaughterhouse by-product	α137–141(TSKYR)	Inhibits the growth of coliform bacteria in meat products	[34]
Vicia faba seeds	LSPGDVLVIPAGYPVAIK, EEYDEEKEQGEEEIR	Antibiofilm activity against *Pseudomonas aeruginosa*	[35]
Hen egg lysozyme	LzP	Inhibits the growth of *B. subtilis*, *B. licheniformis*, *B. megaterium*, *B. mycoides*, *B. pumilus*, *B. coagulans*, *B. amyloliquefaciens*, *B. polymexa* and *B. macerans*	[36]
Bovine milk lactoferrin	LfcinB	Prevents *E. coli* O157:H7 related intestinal dysfunction and also susceptible against *S. enteritidis*, *K. pneumoniae, P. vulgaris, Y. enterocolitica, P. aeruginosa, C. jejuni, S. aureus, L. monocytogenes* and *C. perfringens.*	[37,38]
